# Multidrug resistance in fungi: regulation of transporter-encoding gene expression

**DOI:** 10.3389/fphys.2014.00143

**Published:** 2014-04-16

**Authors:** Sanjoy Paul, W. Scott Moye-Rowley

**Affiliations:** Department of Molecular Physiology and Biophysics, Carver College of Medicine, University of IowaIowa City, IA, USA

**Keywords:** multidrug resistance, transcription, fungal pathogens, ABC transporters, transcription factors, regulation of gene expression

## Abstract

A critical risk to the continued success of antifungal chemotherapy is the acquisition of resistance; a risk exacerbated by the few classes of effective antifungal drugs. Predictably, as the use of these drugs increases in the clinic, more resistant organisms can be isolated from patients. A particularly problematic form of drug resistance that routinely emerges in the major fungal pathogens is known as multidrug resistance. Multidrug resistance refers to the simultaneous acquisition of tolerance to a range of drugs via a limited or even single genetic change. This review will focus on recent progress in understanding pathways of multidrug resistance in fungi including those of most medical relevance. Analyses of multidrug resistance in *Saccharomyces cerevisiae* have provided the most detailed outline of multidrug resistance in a eukaryotic microorganism. Multidrug resistant isolates of *S. cerevisiae* typically result from changes in the activity of a pair of related transcription factors that in turn elicit overproduction of several target genes. Chief among these is the ATP-binding cassette (ABC)-encoding gene PDR5. Interestingly, in the medically important *Candida* species, very similar pathways are involved in acquisition of multidrug resistance. In both *C. albicans* and *C. glabrata*, changes in the activity of transcriptional activator proteins elicits overproduction of a protein closely related to *S. cerevisiae* Pdr5 called Cdr1. The major filamentous fungal pathogen, *Aspergillus fumigatus*, was previously thought to acquire resistance to azole compounds (the principal antifungal drug class) via alterations in the azole drug target-encoding gene *cyp51A*. More recent data indicate that pathways in addition to changes in the *cyp51A* gene are important determinants in *A. fumigatus* azole resistance. We will discuss findings that suggest azole resistance in *A. fumigatus* and *Candida* species may share more mechanistic similarities than previously thought.

## Introduction

Scientifically, one of the most attractive features of the fungal microorganisms is their striking biological similarity with mammalian cells. This similarity has led to their extensive use as models for basic properties of eukaryotic cells but also illustrates one of the important challenges in dealing with fungal pathogens. Development of antifungal drugs has been slowed by the close relationship between the fundamental cell biology of a fungus and a typical mammalian cell. Early antifungal drugs like amphotericin B were efficacious in treating fungal infections but suffer from high toxicity to the patient (Moen et al., 2009).

More recently developed drugs like the azoles and echinocandins, exhibit better specificity for pathogens but their very success has led to the selection of fungi that are highly tolerant of these antifungal agents (recently discussed in Brown et al., 2012). Azole drugs are the primary antifungal drug used in the clinic and infections associated with azole resistant fungi have dramatically lowered survival rates compared to those associated with azole susceptible organisms (reviewed in Pfaller, 2012). Resistance to azole drugs emerges by two different routes: direct and indirect mechanisms. Direct changes involve changes in the enzyme that is targeted by azole compounds, lanosterol 14α demethylase (See Kristan and Rizner, 2012 for a recent review). This enzyme is encoded by the *ERG11* gene in *Saccharomyces cerevisiae* with azole resistant alleles found to either change the sequence of the protein and/or alter expression levels of this gene product. While these direct changes can be found in *S. cerevisiae* and pathogenic yeast, a common form of azole resistance arises via indirect means. Most typically, selection for azole resistant yeast elicits isolation of strains that overproduce ATP-binding cassette (ABC) transporter proteins. These ABC transporter proteins act to efflux azole drugs from the cell and prevent accumulation of otherwise toxic levels (recently reviewed in Prasad and Goffeau, 2012).

ABC transporter overproduction is a well-known cause of drug resistance in mammalian cells (See Holohan et al., 2013 for a recent discussion). Cancer patients undergoing chemotherapeutic treatment are often found to eventually develop tumors that are termed multidrug resistant. These *in vivo* selected tumors are refractory to growth inhibition caused by a wide range of different chemotherapeutics, even drugs to which the patient was not previously exposed (Szakacs et al., 2006). Multidrug resistant tumors can be associated with gene amplification events that lead to an increase in copy number of genes encoding ABC transporters with a concomitant elevation in protein level (Ambudkar et al., 2003).

In fungal cells, multidrug resistant cells can easily be selected. These mutant strains do not usually have increased gene dosage but rather possess increased expression of ABC transporter-encoding genes (reviewed in Sanglard et al., 2009; Morschhauser, 2010). Elevated gene expression leads to an increase in the protein level of these membrane transporters with accompanying broad range drug tolerance. In every fungus that has been studied, ABC transporters are able to influence azole resistance making this a key issue in treatment of fungal infections.

Given the importance of the level of ABC transporter gene expression in development of azole resistance, their regulation has been the focus of extensive investigation. These studies have led to the identification of dedicated transcription factors dedicated to coordination of their expression with other genes in fungi. Our most detailed picture of the network of transcriptional regulation and multidrug resistance gene is available for the yeast *Saccharomyces cerevisiae*. This network has served as a starting point for the analysis of similar systems in pathogenic fungi and will be discussed below.

## Transcriptional circuitry controlling pleiotropic drug resistance in *S. cerevisiae*

### Zn_2_Cys_6_ zinc cluster-containing factors

The first transcription factor discovered to be a determinant of multidrug resistance in *S. cerevisiae* was appropriately designated Pdr1 (Saunders and Rank, 1982). Early genetic work pointed to the existence of dominant alleles of the *PDR1* gene as conferring resistance to a wide range of different toxic compounds (Rank and Bech-Hansen, 1973; Rank et al., 1975, 1976). This broad range resistance phenotype in *S. cerevisiae* was designated pleiotropic drug resistance (Pdr) and is functionally analogous to the multidrug resistance seen in mammalian cells and other fungi. This early work has been the subject of several reviews (Balzi and Goffeau, 1995; Bauer et al., 1999; Gulshan and Moye-Rowley, 2007) and will only be outlined here.

Isolation of the cloned *PDR1* gene by Balzi et al. (1987) led to rapid advances in understanding of the molecular basis of the Pdr phenotype in *S. cerevisiae*. The DNA sequence of *PDR1* led to two important findings. First, this gene encoded a Zn_2_Cys_6_ cluster-containing transcription factor similar to the previously described Gal4 (Johnston, 1987). Second, sequence of a dominant, multidrug resistant allele of *PDR1* demonstrated that this hypermorphic or gain-of-function (GOF) behavior of Pdr1 was caused by a single amino acid substitution mutation in the central region of the protein. Many different amino acid substitution mutations were eventually discovered that caused Pdr1 to exhibit elevated, constitutive transcriptional activation of target gene expression (Carvajal et al., 1997).

The discovery that Pdr1 was a transcriptional regulator led to work from multiple laboratories to identify genes that were more directly responsible for the observed multidrug resistance phenotype. The first major class of Pdr1 target genes contained several different loci encoding ABC transporter proteins (Balzi et al., 1994; Bissinger and Kuchler, 1994; Hirata et al., 1994a; Katzmann et al., 1995; Mahe et al., 1996). These proteins were typically found in the plasma membrane and thought to act as broad specificity, drug efflux pumps as previously seen in mammalian tumor cells (Gottesman et al., 1995). The *PDR5* locus was found to possess unique central importance among the ABC transporter-encoding genes as the expression level of this gene is the highest of the drug resistance-related ABC transporters and mutants lacking this gene exhibit profound drug hypersensitivities (Leppert et al., 1990; Decottignies et al., 1994). *PDR5* transcription was strongly elevated in cells containing hyperactive *PDR1* dominant alleles through the binding of the transcriptional regulatory protein to three sites in the promoter region (Katzmann et al., 1996). These sites are referred to as Pleiotropic Drug Response Elements or PDREs and have been found upstream of the majority of Pdr1-regulated genes.

A factor closely related to Pdr1 was discovered during genome sequencing experiments and designated *PDR3* (Delaveau et al., 1994). GOF *PDR3* alleles similar to those described for *PDR1* were isolated and elevated *PDR5* transcription and drug resistance found to occur in these strains (Dexter et al., 1994; Nourani et al., 1997; Simonics et al., 2000). Generally speaking, the phenotypic effects of activated alleles of *PDR1* and *PDR3* are very similar. However, a feature unique to the *PDR3* gene was the presence of two PDREs in its promoter (Delahodde et al., 1995). This autoregulatory input was found to be essential for normal function of *PDR3* and is a key component for the regulated expression of this gene (Delahodde et al., 1995; Zhang and Moye-Rowley, 2001).

Along with Pdr1 and Pdr3, several other zinc cluster-containing transcription factors have been associated with the Pdr phenotype. The first of these, Yrr1, was recovered in a screen for tolerance to a cell cycle inhibitor called reveromycin A (Cui et al., 1998). Later work indicated that Yrr1 was both autoregulated and transcriptionally induced by Pdr1/Pdr3 activity (Zhang et al., 2001). Several laboratories discovered that *YRR1* mutants could be recovered that exhibited high constitutive transcriptional activation as previously discussed for similar alleles in *PDR1* and *PDR3* (Cui et al., 1998; Zhang et al., 2001; Keeven et al., 2002; Le Crom et al., 2002). Two homologs of Yrr1 have also been studied: Yrm1 (Lucau-Danila et al., 2003) and Pdr8 (Hikkel et al., 2003).

### Non-transporter targets genes of PDR1/PDR3

While the first genes identified that were controlled by the Pdr regulon were ABC transporters, more recent data make it clear that many different types of proteins are encoded by Pdr1/Pdr3 target genes (Derisi et al., 2000; Devaux et al., 2001, 2002; Traven et al., 2001). These other classes of proteins include 7 transmembrane domain-containing membrane proteins (Rsb1, Rta1) (Kihara and Igarashi, 2004; Panwar and Moye-Rowley, 2006; Kolaczkowska et al., 2012), enzymes involved in sphingolipid biosynthesis (Hallstrom et al., 2001; Kolaczkowski et al., 2004), phospholipid transfer (Van Den Hazel et al., 1999), and other proteins of poorly characterized function. Given the roles for many of these other proteins in lipid homeostasis it is reasonable to propose that the control of lipids has an important effect on drug resistance. Evidence in support of this notion is accumulating in S. cerevisiae and other fungi (Hallstrom et al., 2001; Kolaczkowski et al., 2004; Prasad et al., 2005). While it is important to recognize that the Pdr response involves more than simply transporter genes, these are the best understood in terms of their role in drug resistance. We will focus on the control of transporter genes in this review.

### Basic region-leucine zipper-containing transcription factors

One of the earliest transcriptional regulators found to influence Pdr was the basic region-leucine zipper (bZip)-containing factor Yap1. Yap1 was isolated in a high-copy-plasmid suppressor screen as *PDR4*, a gene capable of elevating resistance to cycloheximide and the branched chain amino acid biosynthesis inhibitor sulfometuron methyl (Leppert et al., 1990). Later work demonstrated that *YAP1* and *PDR4* were allelic (Hussain and Lenard, 1991) and that Yap1 did not appear to act via *PDR5* (Dexter et al., 1994). Work from several labs demonstrated that the bZip-containing protein Cad1/Yap2 also elevated drug resistance although this effect could only be seen when this protein was overproduced (Bossier et al., 1993; Wu et al., 1993; Hirata et al., 1994b). Similarly, high dosages of the Yap1 homologs Yap5 and Yap6 also conferred resistance to several different drugs (Fernandes et al., 1997; Furuchi et al., 2001).

While there are clear links between the bZip-containing factors and drug resistance, most of the drug resistance effects shown by this group of transcription factors occur when these proteins are overproduced. Disruption mutations rarely exhibit hypersensitivity suggesting either that extensive redundancy exists between these family members or that the normal role of these factors is not to modulate drug resistance. Yap1 is the best understood of these bZip-containing factors in terms of its regulation (reviewed in Toone et al., 2001; Moye-Rowley, 2003a; Rodrigues-Pousada et al., 2004). Loss of the *YAP1* gene has a profound sensitivity to oxidative stress (Schnell et al., 1992; Kuge and Jones, 1994) but little to no consequence on drug resistance (Leppert et al., 1990; Wu et al., 1993; Alarco et al., 1997). An exception to this rule is provided by drugs such as the agrochemical mancozeb that proceed via an oxidative mechanism in which physiological levels of Yap1 are required for normal resistance (Teixeira et al., 2008). Drugs that act through a mechanisms that does not invoke an oxidative stress response typically are insensitive to the presence of Yap1 expressed at normal gene dosage. Yap1 has been thoroughly documented as an important regulator of major facilitator superfamily (MFS) gene expression in the case of *FLR1* (Alarco et al., 1997) and *ATR1* (Coleman et al., 1997). Activation of expression of these genes is likely to explain many of the effects of this transcription factor on drug resistance although little evidence exists that Yap1-dependent transactivation can be stimulated by the presence of drugs. Extensive documentation exists for activation of Yap1 function upon oxidative stress (Kuge et al., 1997; Coleman et al., 1999; Delaunay et al., 2000, 2002). We will focus our discussion on the Zn_2_Cys_6_ zinc cluster-containing proteins as the paradigms for drug-induced transcriptional activation in *S. cerevisiae* (and likely other fungi as well).

### Control of transcriptional activation in *S. cerevisiae* multidrug resistance

**Figure 1:**
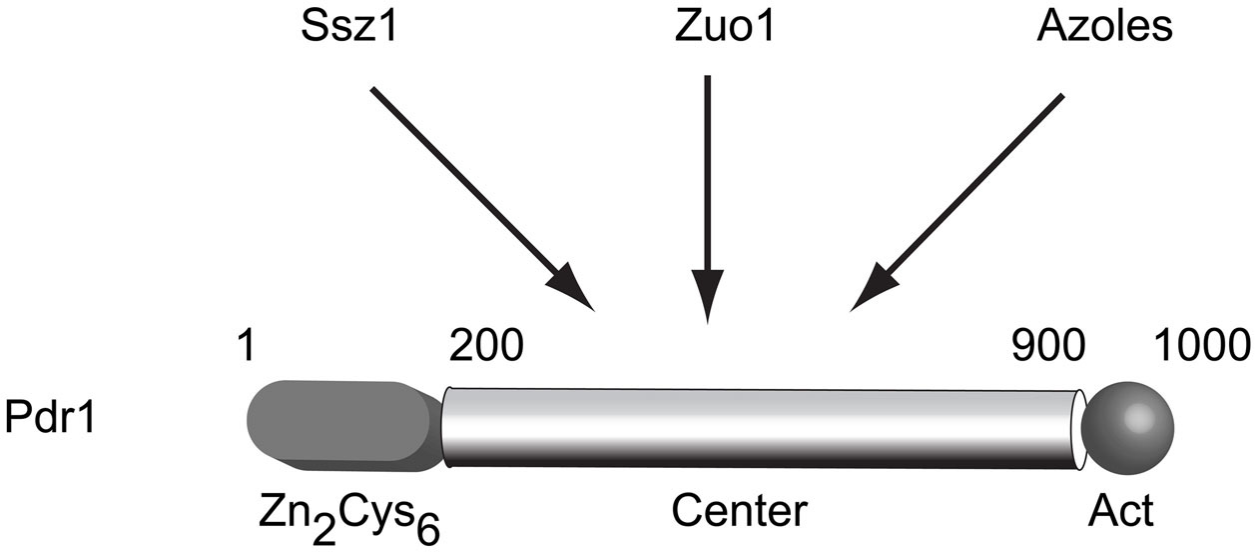
**Structure and regulation of *S. cerevisiae* Pdr1**. A cartoon of the predicted structure of the *S. cerevisiae* Pdr1 transcription factor is shown. The amino-terminal DNA binding domain containing a Zn_2_Cys_6_ cluster is located between amino acids 1–200 while the transcriptional activation domain lies at the C-terminus of the protein spanning approximately residues 900–1000. The presumptive regulatory domain that is targeted by several stimulatory signals is referred to as the center domain and lies between residues 200–900. The center domain is thought to be independently regulated by the Hsp70 protein Ssz1, the dnaJ protein Zuo1 and by direct binding to xenobiotics (illustrated here by azole drugs).

The identification of alleles of *PDR1* as dominant, GOF mutations suggested the possibility of regulatory modulation of the Pdr1 protein. The determination that Pdr1 and Pdr3 were closely related Zn_2_Cys_6_ zinc cluster-containing transcription factors also argued that these proteins were most likely to be indirect determinants of multidrug resistance via their regulation of direct effectors of this phenotype (such as *PDR5*). Sequence analysis of a large number of these hyperactive mutant alleles of *PDR1* and *PDR3* demonstrated that this multidrug resistant phenotype was routinely caused by single amino acid substitutions in three main regions of these factors (Dexter et al., 1994; Carvajal et al., 1997; Nourani et al., 1997; Simonics et al., 2000). A model of Pdr1 structure is shown in Figure 1. Early analyses of these GOF alleles indicated that target genes were overexpressed in the presence of the mutant forms of the transcription regulators. For example, *PDR5* mRNA levels are increased by roughly 10-fold in the presence of a *PDR1* GOF allele compared to levels driven by the wild-type gene (Meyers et al., 1992). This correlates well with the observed increment in drug resistance.

While the characterization of the GOF mutants supported the view that the factors regulating Pdr were under some type of regulatory control, these alleles represent chronic functional alterations in these transcription factors. Here we will summarize more recent progress in elaboration of mechanisms controlling activity of either wild-type Pdr1 or Pdr3. To date, regulatory inputs to these two closely related proteins are unique to each factor and will be considered separately.

### PDR1 regulation

The first example of a trans-factor influencing Pdr1 activity came from the identification of the Hsp70 protein Ssz1 (originally Pdr13) as a positive regulator of Pdr1 function when overproduced (Hallstrom et al., 1998). Later work demonstrated that this Hsp70 was primarily associated with the ribosome (Hallstrom and Moye-Rowley, 2000a) and has an important role in folding of nascent polypeptide chains (Gautschi et al., 2001). Recently, these findings have been extended with the determination that the dnaJ protein Zuo1 (partner of the dnaK Ssz1 Michimoto et al., 2000) can also stimulate Pdr1 function, possibly by direct binding to this transcription factor (Eisenman and Craig, 2004). Zuo1 contains a short C-terminal region of 13 residues that is necessary and sufficient to induce Pdr1 transcriptional activity (Ducett et al., 2013).

Both Zuo1 and Ssz1 are present at much higher levels than Pdr1 and are thought to have their primary function at the ribosome (Gautschi et al., 2001). Work from the Craig lab has argued that the Zuo1/Ssz1 protein pair can independently activate Pdr1 and are found directly associated with this factor on target promoters (Prunuske et al., 2012). As these findings suggest, the action of Zuo1/Ssz1 in terms of control of Pdr1 activity represents an extra-ribosomal function of these proteins (Eisenman and Craig, 2004). Despite significant effort, the molecular rationale linking activity of these chaperone proteins to Pdr1 remains obscure. An interesting possible tie between Pdr1 and protein folding/degradation came from the finding that the gene encoding the key proteasomal transcriptional regulator Rpn4 is a transcriptional target of Pdr1 (Devaux et al., 2001; Owsianik et al., 2002). Activation of Pdr1 function by Ssz1 can suppress defects in endoplasmic reticulum-associated degradation (Bosis et al., 2010) further strengthening the association of Pdr1 activity with protein homeostasis.

An intriguing model for Pdr1 (and Pdr3) to act as xenobiotic receptor proteins came from studies led by Anders Näär (Thakur et al., 2008). This work provided evidence that the central regions of both these transcription factors could directly bind radioactive fluconazole and that this antifungal drug triggered an increase in Pdr1/3-dependent gene expression. These data support the attractive notion that Pdr1/3 were normally held in a low activity state but could be remodeled in the presence of an appropriate ligand to a more potent transcriptional stimulator.

The simplicity and precedence of this model make it a compelling view for the regulation of Pdr1/3 in physiology. It is still difficult to rationalize why overproduction of either Pdr1 or Pdr3, in the absence of any exogenous drug, can lead to increased gene expression (see for example: Katzmann et al., 1994; Carvajal et al., 1997). If activation of these factors required the presence of a small molecule, then overproduction would not be expected to lead to increase downstream gene expression. In cases where a small molecule is known to be required for activity of a zinc cluster-containing transcription factor, overproduction of the factor leads to downstream repression of gene expression. Examples of this behavior include the heme-dependent activator Hap1 (Zhang et al., 1998) and the leucine biosynthetic activator protein Leu3 (Sze et al., 1993). The genetic behavior of Pdr1/3 seem more easily fit to a situation in which a negative regulatory system is outcompeted when these proteins are overproduced. Further experiments are required to dissect the mechanism of Pdr1/3 control via by small molecules.

### PDR3 regulation

Although hyperactive alleles of *PDR3* were identified at the inception of the study of the Pdr network in *S. cerevisiae* (Dexter et al., 1994), early analyses of the contribution of Pdr3 to drug resistance suggested that this factor was a less important contributor to transcriptional control than its homolog Pdr1 (Delaveau et al., 1994; Katzmann et al., 1994). Screening a library of random transposon-induced null mutations to identify negative regulators of *PDR5* expression uncovered an array of gene involved in maintaining the mitochondrial genome (Hallstrom and Moye-Rowley, 2000b). Loss of mitochondrial DNA (ρ^0^) led to a strong induction of *PDR5* gene expression that was strictly Pdr3-dependent. This was the first example of a situation in yeast in which the level of *PDR5* expression and associated drug resistance rivaled those seen in the presence of a hyperactive allele of either *PDR1* or *PDR3*.

**Figure 2:**
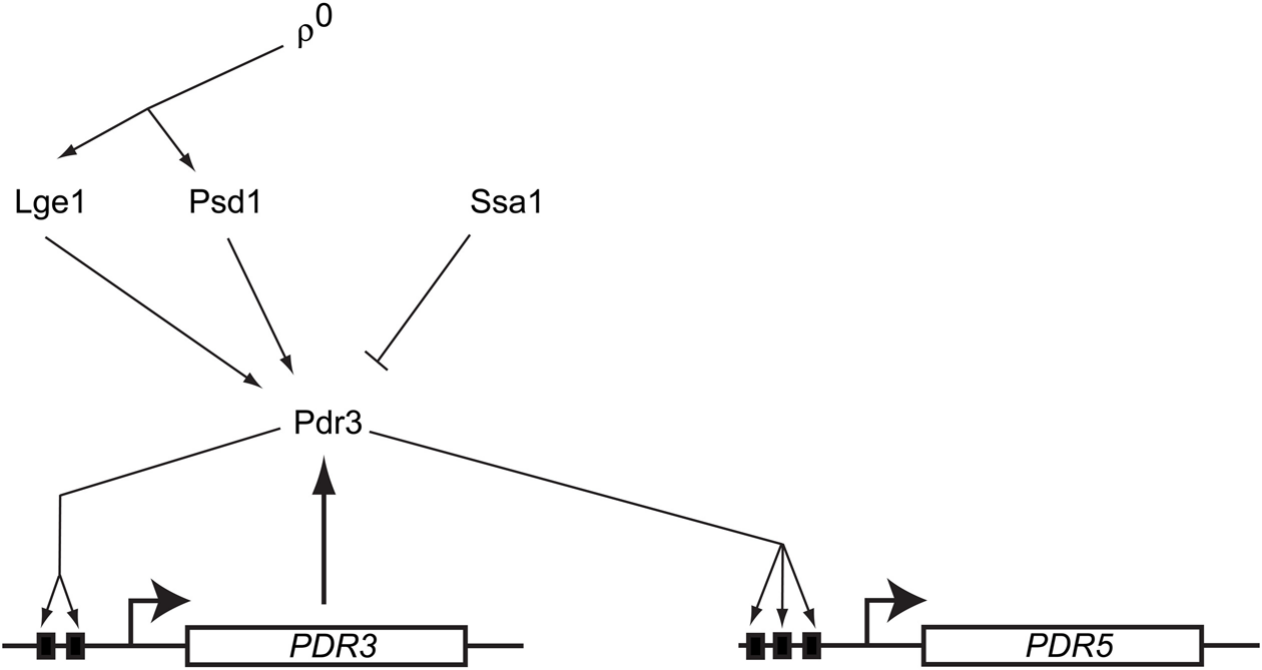
**Regulation of *S. cerevisiae* Pdr3**. A diagram showing the regulatory inputs modulating Pdr3 function is presented. Loss of mitochondrial DNA (ρ^0^) signals move through the PE carboxylase protein Psd1 and via the nuclear factor Lge1. Azole drugs have been demonstrated to directly bind to the center region of Pdr3. The Hsp70 protein Ssa1 associates with and represses activity of Pdr3. The PDR3 gene is under positive autoregulation via the presence of two Pleiotropic Drug Response Elements (PDREs) depicted as solid black boxes. Pdr3 also activates transcription of downstream genes like *PDR5* that confer the multidrug resistance phenotype.

Characterization of the requirements for this mitochondrial signal to trigger Pdr3 induction demonstrated that of the many types of mutations compromising mitochondrial function, only those that also elicited loss of mitochondrial DNA were able to elevate Pdr3 function (Zhang and Moye-Rowley, 2001). The autoregulatory loop of *PDR3* transcription was also essential for the ρ^0^ response. However, driving Pdr3 expression from the *PDR1* promoter was still able to produce a ρ^0^-dependent induction in drug resistance (Hallstrom and Moye-Rowley, 2000b) arguing that Pdr3 was regulated post-translationally. Together, these findings suggest that a mitochondrial genome-dependent signal leads to a release of Pdr3 from some negative regulatory system. This in turn allows Pdr3 to engage with its own promoter and positively autoregulate transcription. Pdr3 regulation is diagrammed in Figure 2.

Further genetic analyses identified several other factors that are required for the normal mitochondrial control of Pdr3. A protein of unknown function called Lge1 was identified as a participant in ρ^0^-induced gene expression (Zhang et al., 2005). Lge1 is involved in ubiquitination of histone H2B (Hwang et al., 2003) but this function seems to be distinct from its role in ρ^0^ induction of *PDR5* expression (Zhang et al., 2005). Mutants lacking Lge1 fail to fully induce *PDR5* transcription in ρ^0^ cells. Overproduction of an enzyme involved in biosynthesis of phosphatidylethanolamine (PE) was discovered to increase Pdr3-dependent transactivation even in ρ^+^ cells (Gulshan et al., 2008). This enzyme, called Psd1, is one of two enzymes typically used to produce PE and is located in the inner membrane space of mitochondria (reviewed in Voelker, 1997). Surprisingly, overproduction of a catalytically-inactive form of Psd1 was still capable of inducing *PDR5* expression even though this mutant enzyme could no longer carry out PE biosynthesis (Gulshan et al., 2008). The presence of Psd1 was required for normal ρ^0^ signaling to occur consistent with this enzyme having a role in wild-type signal transduction.

Mass spectrometric analysis of purified Pdr3 led to the identification of the Hsp70 protein Ssa1 as a negative regulator of this transcription factor (Shahi et al., 2007). Overproduction of Ssa1 caused a decrease in Pdr3-dependent gene expression and hyperactive mutants exhibited less binding to Ssa1 *in vivo* than wild-type Pdr3. This reduction in Hsp70 association correlated with increased transcriptional activation capability is reminiscent of the regulation of steroid hormone receptors (Pratt and Toft, 1997); however, the presence of other heat shock proteins docked to Pdr3 has not been reported. Interestingly, both Pdr1 and Pdr3 have been reported to be induced by challenge with progesterone, furthering the analogy with a regulatory mechanism resembling steroid hormone receptors (Banerjee et al., 2008).

Further biochemical experiments seeking to identify interacting proteins capable of binding to the KIX domain of the transcriptional Mediator subunit Med15/Gal11 determined that both Pdr1 and Pdr3 interacted with this protein region (Thakur et al., 2008). The Mediator complex is composed of roughly 20 different proteins that act to provide a link between sequence-specific transcriptional regulatory proteins and the RNA polymerase II machinery (see Casamassimi and Napoli, 2007 for a review). Biochemical and genetic experiments have separated the Mediator complex into two different forms referred to as either S-Mediator (or core mediator) containing three and L-Mediator containing four subdomains (Poss et al., 2013). The three subdomains of S-Mediator are called Head, Middle, and Tail with Med15 contributing a component of the Tail complex of proteins (Beve et al., 2005). L-Mediator contains an additional subcomplex consisting of the cyclin-dependent kinase Cdk8 (or Med15/Srb8) and attendant cyclin: cyclinC (Srb11) (reviewed in Conaway and Conaway, 2011). Two other large proteins designated Med12 (Srb8) and Med13 (Srb9) complete the Cdk8 complex.

Interactions between Pdr3 (and Pdr1) and Med15 are crucial for normal levels of downstream gene expression under all conditions (Shahi et al., 2010). However, activation of Pdr3 function in ρ^0^ cells still occurs in *med15*Δ cells. Loss of the Med12 protein from the Cdk8 complex completely blocked ρ^0^ induction of *PDR5* transcription but had a negligible effect on expression in ρ^+^ cells. These findings argue that Med12 is a key target for activated transcription supported by Pdr3, at least in ρ^0^ cells. Generally speaking, the Cdk8 complex is thought to serve repressive functions in terms of transcriptional control but more recent data argue that this complex also has positive influences on gene expression as illustrated by the case of Pdr3 activation in ρ^0^ cells.

## Multidrug resistance in *Candida albicans*

The major human fungal pathogen is the yeast *Candida albicans*. This organism, while susceptible to azole drug treatment, is readily detected to acquire a multidrug resistance phenotype that includes robust tolerance to this important class of antifungal drug (reviewed in Pfaller, 2012). Next to the current picture of *S. cerevisiae* multidrug resistance, the situation in *C. albicans* is the best understood and has been the focus of authoritative reviews (Cannon et al., 2009; Sanglard et al., 2009; Morschhauser, 2010). Here we will focus our attention on the transcriptional regulatory proteins implicated in clinically relevant resistance phenotypes.

### Membrane proteins important in multidrug resistance in *C. albicans*

The two best characterized genes involved in multidrug resistance in C. albicans are the ABC transporter-encoding *CDR1* gene (Prasad et al., 1995) and the MFS protein-encoding *MDR1* locus (Fling et al., 1991). Both of these resistance determinants are localized to the plasma membrane in *C. albicans* (Manoharlal et al., 2008; Basso et al., 2010; Kapoor et al., 2010). *CDR1* was isolated on the basis of complementation of the drug hypersensitivity of a *pdr5*Δ mutant in *S. cerevisiae*. Loss of *CDR1* led to a pronounced drug sensitive phenotype in *C. albicans*. A closely related ABC transporter-encoding gene, designated *CDR2*, also contributes to drug resistance when overproduced or when *CDR1* is deleted (Sanglard et al., 1997).

*MDR1* has been documented to be overexpressed in a number of different clinical isolates (Morschhauser, 2002); however, its loss does not appear to have significant effects on baseline drug resistance. The plasma membrane location of these transporter proteins supports the view that these proteins act as efflux pumps for a range of different drugs (Schuetzer-Muehlbauer et al., 2003; Lettner et al., 2010). Multidrug resistance in *C. albicans* is associated in large part with transcriptional induction of the genes encoding these membrane proteins.

### TAC1

The first insight into the molecular basis of transcriptional regulation of multidrug resistance in *C. albicans* came from the identification of the *TAC1* locus. As with *PDR1* and *PDR3* in *S. cerevisiae*, genetic observations drove the discovery of the *TAC1* gene. Analyses of azole tolerant *C. albicans* isolates led to the discovery of loss of heterozygosity/aneuploidy around the mating type loci (MTL) gene cluster (Rustad et al., 2002). Using this observation as a starting point, Sanglard and colleagues took a candidate gene approach by disrupting several different Zn_2_Cys_6_ zinc cluster-encoding genes in this chromosomal region (Coste et al., 2004). Loss of one of these genes, designated *TAC1* (Transcriptional Activator of Cdr genes), resulted in a strain displaying enhanced azole susceptibility and depressed expression of the *CDR1* ABC transporter-encoding gene.

Along with a requirement for *TAC1* to confer normal wild-type expression of *CDR1* and drug resistance, evidence was obtained that two different changes in the *TAC1* gene influenced its function. First, a number of different substitution and even small deletion mutations strongly enhanced the function of Tac1 (Coste et al., 2007). Secondly, chromosomal rearrangements of chromosome 5 were detected that led to loss of heterozygosity and changes in the dosages of the linked *ERG11* and *TAC1* genes. These structural changes in chromosome 5 were found to lead to the amplification and likely overexpression of *ERG11* and *TAC1* (Coste et al., 2007; Selmecki et al., 2008). These findings are consistent with a model in which Tac1 can be activated either by changes in its primary sequence or simply by overproducing the wild-type factor. Consistent with the notion that increased expression of Tac1 leads to increased function comes from data that the *TAC1* gene is positively autoregulated (Liu et al., 2007; Znaidi et al., 2007).

Although we do not yet know the nature of the signals that control Tac1, the currently available data suggest that its regulation may resemble that of Pdr3 in *S. cerevisiae*. Both of these factors can be activated by mutations along their respective primary sequence and the genes encoding these regulators are positively autoregulated.

### Mrr1

A second zinc cluster-containing transcription factor that is an important contributor to drug resistance was identified in cells that were already known to overproduce *MDR1* (Morschhauser et al., 2007). Transcriptional profiling experiments demonstrated that fluconazole resistant isolates shared a common overproduced transcript encoding a zinc cluster-containing transcription factor. This regulatory protein was designated Mrr1 (multidrug resistance regulator). The presence of Mrr1 is required for the observed overproduction of *MDR1* in fluconazole resistant strains. While the *MDR1* promoter contains binding sites for other positive regulators, loss of Mrr1 is sufficient to abrogate the overproduction that occurs in drug resistant isolates. This argues that Mrr1 is the key transcriptional regulator during development of drug resistance involving *MDR1* overproduction (Dunkel et al., 2008). Importantly, azole resistant clinical isolates have been described that overproduce *MDR1* and are dependent upon the presence of this MFS protein for this drug resistance (White, 1997; Wirsching et al., 2000).

Genetic activation of Mrr1 shows similar behavior to other zinc cluster-containing transcription factors in which single amino acid substitutions lead to dramatic induction of Mrr1 transcriptional activity (Dunkel et al., 2008). Detailed analyses of the functional subdomains within Mrr1 indicate that the regulation of this factor is likely to be complex with separable inhibitory and activation domains (Schubert et al., 2011b). Chromatin immunoprecipitation experiments also indicated that Mrr1 is likely to control its own expression (Schubert et al., 2011a) as mentioned above for Tac1. Both these *C. albicans* factors appear to share common overall features with ScPdr3 that is also autoregulated and can be genetically activated by a range of different mutations (reviewed in Moye-Rowley, 2003b).

Along with their important effects on multidrug resistance and critically azole tolerance, these transcription factors have been examined for their influence on virulence. Systematic introduction of GOF forms of *TAC1* and *MRR1* indicated a fitness cost in a gastrointestinal colonization model caused by either mutant gene individually that was exacerbated with their combination (Sasse et al., 2012). These authors suggested an interesting potential rationale for the common observation that clinical isolates of azole resistant *C. albicans* contain either *TAC1* or *MRR1* mutations but not both. Although the double *TAC1 MRR1* strains were robustly azole tolerant, the fitness cost could be so great as to exclude this genetic combination *in vivo*. More recent data suggests that certain combinations of azole resistance mechanisms, including simultaneous presence of mutations in *TAC1* and *MRR1*, may be tolerated (Morio et al., 2013).

A different infection model was used to investigate similar mutations in these same transcription factor genes (tail vein injection) (Lohberger et al., 2014). Using this bloodstream infection model, no significant fitness defect was found consistent with the interpretation that, at least under certain conditions, GOF forms of these transcription factors could be well-tolerated *in vivo*.

### Other transcription factors involved in drug resistance

The intensive investigation of drug resistance in *C. albicans* has led to identification of a range of different transcriptional regulatory proteins that participate at some level in drug resistance including Ndt80 and Mcm1 among others. These factors in general have not been associated with clinically-relevant drug resistance and seem to play roles as expression modifiers of target genes involved in azole resistance (Mogavero et al., 2011; Sasse et al., 2011).

Two other transcription factors have been linked to certainly azole resistance if not multidrug resistance in *C. albicans*. The Upc2 transcriptional regulator is a positive modulator of sterol biosynthesis and mutations in this factor lead to pronounced azole resistance (Flowers et al., 2012). The effects of Upc2 on drug resistance occur via its role as a key regulator of sterol biosynthesis making this factor unlikely to serve as a true multidrug resistance determinant. *CAP1* encodes the *C. albicans* ScYap1 homolog but does not appear to participate in drug resistance when expressed at normal levels. (Alarco et al., 1997; Alarco and Raymond, 1999). Cap1 clearly regulates *MDR1* (Rognon et al., 2006) but this may have more to do with a role for Mdr1 in the oxidative stress response than drug resistance.

## Multidrug resistance in *C. glabrata*

*Candida glabrata* is the second most common cause of bloodstream and mucosal candidiasis (10–30%) in the United States, with a high mortality rate (38–53%) (Richter et al., 2005; Pfaller and Diekema, 2007). *C. glabrata* is intrinsically resistant to fluconazole, a common azole drug that targets the fungal specific ergosterol biosynthetic pathway, with resistance known to further increase during drug therapy (8–27% of isolates demonstrating a fluconazole MIC ≥ 64 μ g ml^−1^) (Ostrosky-Zeichner et al., 2003; Pfaller et al., 2004). As for other fungi, *C. glabrata* azole resistance can be the result of an alteration of the target enzyme by either overexpression or mutations in its encoding gene, *ERG11*, that reduces the efficacy of the drug (Henry et al., 2000).

Another frequent basis for multiazole tolerance phenotype is enhanced drug efflux mediated by the activation of expression of ABC transporters like *CgCdr1*, *CgCdr2* (*Pdh1*), and *CgSnq2* (Sanglard et al., 1995, 1997; Miyazaki et al., 1998; Redding et al., 2003; Bennett et al., 2004; Vermitsky and Edlind, 2004; Sanguinetti et al., 2005; Torelli et al., 2008). CgCdr1 seems to be the chief ABC transporter that is constitutively upregulated in most of the drug resistant clinical isolates, either by itself, or as a combination with *CgCdr2* (*Pdh1*) and/or *CgSnq2*. Deletion of *CgCdr1* leads to increased intracellular azole accumulation and hypersensitivity to different azoles, both in clinical isolates as well as lab strains. The ABC transporters mentioned above are regulated by a Zn_2_Cys_6_ zinc cluster-containing transcription factor encoded by *CgPDR1*. It is now well established that *CgPDR1* exhibits GOF mutations in most of the drug resistant clinical isolates of *C. glabrata* leading to upregulation of the transcription factor, which is responsible for high expression of the target genes that encode drug efflux pumps mediating multidrug resistance. It has been demonstrated that some of these *CgPDR1* GOF mutations also lead to hypervirulence in a mouse model for systemic candidiasis (Ferrari et al., 2009). Interestingly, further work from this same group found that CgPdr1 regulated ABC transporter CgCdr1 and a mitochondrial protein, Pup1, is at least partially responsible for this gain of virulence (Ferrari et al., 2011b).

### CgPdr1

**Figure 3:**
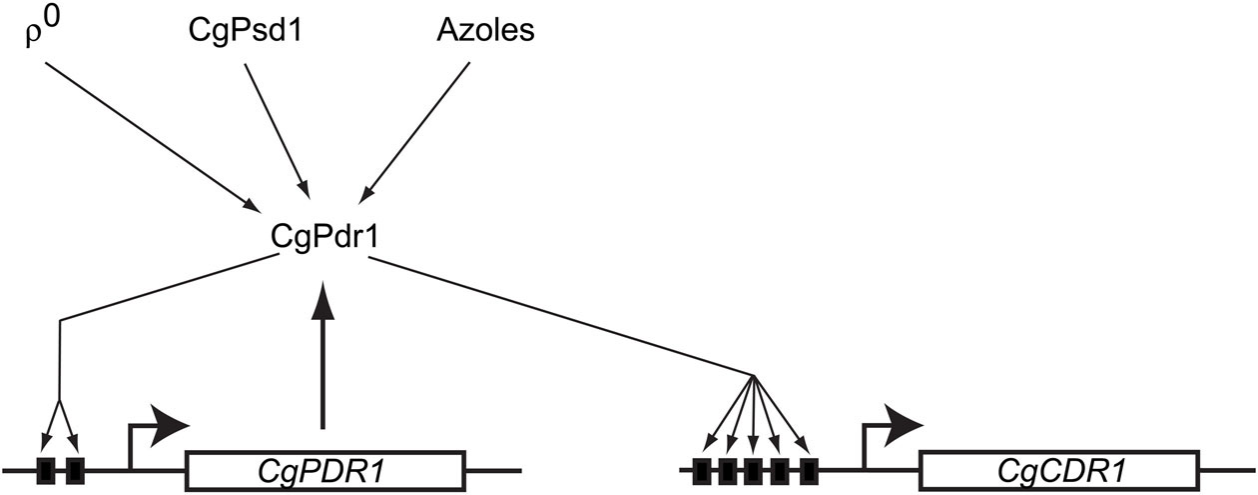
**Regulation of multidrug resistance gene expression in *Candida glabrata***. A model reflecting the currently identified players controlling CgPdr1 activity and multidrug resistance gene expression is shown. The solid black boxes indicate the PDREs in the promoters of *CgPDR1* and *CgCDR1*. To date, only signals that positively regulate CgPdr1 have been identified. Note the similarities with ScPdr3 regulation.

Consistent with the fact that *C. glabrata* is phylogenetically closer to *S. cerevisiae* than *C. albicans*, the multidrug resistance pathways in *S. cerevisiae* and *C. glabrata* share more extensive similarities than with the cognate *C. albicans* system. The molecular players involved in drug resistance, ρ^0^-induced PDR pathway activation, and the fact that the pivotal control of multidrug resistance in *C. glabrata* is at the transcriptional level are all shared with *S. cerevisiae*. However, there are interesting differences as well. Unlike *S. cerevisiae*, *C. glabrata* ABC transporters involved in multidrug resistance is regulated by the single transcription factor CgPdr1. CgPdr1 seems to be a blend of transcription factors ScPdr1 and ScPdr3 that modulate drug resistance in *S. cerevisiae*. Though CgPdr1 shares greater identity with ScPdr1 in terms of protein sequence, the *C. glabrata* transcription factor has binding elements in its own promoter contributing to its autoregulation, just as in the case of ScPdr3. Also like ScPdr3, CgPdr1 responds to Pdr pathway activation in ρ^0^ cells that lack mitochondrial DNA, as well as when the mitochondrial enzyme phosphatidylserine decarboxylase (ScPsd1) is overproduced (Paul et al., 2011). The role played by the Mediator component Gal11 in regulation of CgPdr1 is also interestingly different in *C. glabrata* when compared to its cognate role in *S. cerevisiae*. There are 2 *GAL11* paralogs (*CgGAL11A* and *CgGAL11B*) in *C. glabrata*, of which only *CgGAL11A* seems to be important for CgPdr1 induced drug resistance (Thakur et al., 2008). The requirement for CgGal11A is however seen only in ρ^+^ and not in ρ^0^ cells, unlike in *S. cerevisiae* (Paul et al., 2011). Moreover, CgGal11A is required for induction of drug resistance upon azole challenge in ρ^+^ cells (Figure 3). This phenomenon of xenobiotic/drug-induced efflux pump activation, while not consistently seen in *S. cerevisiae*, is similar to that observed in *C. albicans* (Liu et al., 2005).

*C. glabrata* respiratory deficient mutants (ρ^0^) that have been generated *in vitro* by either ethidium bromide or azole treatment (where ρ^0^ cells occur at high frequency), exhibit a growth defect phenotype and reduced virulence (Brun et al., 2005). However, these same cells also possess robust drug resistance that is primarily based on activation of the transcription factor CgPdr1 and upregulation of its target gene CgCdr1 (Vermitsky and Edlind, 2004; Tsai et al., 2006). Interestingly, such mutants have, albeit rarely, been recovered from patients undergoing fluconazole treatment, suggesting that this mechanism of drug resistance may be clinically relevant (Bouchara et al., 2000). Recently, it was reported that an azole resistant *C. glabrata* ρ^0^ isolate from the clinic (containing a wild-type *CgPDR1* gene) exhibited higher virulence and *in vivo* fitness in a murine infection model than its azole-susceptible and respiration-competent parental strain (Ferrari et al., 2011a).

Intrinsic low susceptibility and high frequency of resistance to widely used azole antifungals have led to greater use of other drugs, particularly amphotericin B and echinocandins in treatment of candidemias involving *C. glabrata* (Sanguinetti et al., 2005). Echinocandins such as caspofungin and micafungin are lipopeptide inhibitors of β-1,3-glucan synthase and interfere with fungal specific cell wall synthesis. This class of antifungals is active against most *Candida* species, including *C. glabrata* (Ostrosky-Zeichner et al., 2003; Pfaller et al., 2008) and has been used as first-line therapy against *C. glabrata* infections (Pappas et al., 2009). Echinocandin resistance in *C. glabrata* is rare by comparison to azole tolerance but increasing in frequency in response to increasing clinical use (Pfaller et al., 2012). Echinocandin resistance maps to genes encoding components of the β-1,3-glucan synthase (recently discussed in Alexander et al., 2013) and as such is considered outside the typical multidrug resistance determinants in this organism.

To overcome the intrinsic and high frequency acquisition of azole resistance in *C. glabrata*, alternative therapies have also been employed. As mentioned above, echinocandins are effective antifungal agents against *C. glabrata* but resistance to these drugs is on the rise. Combined therapies have also been explored but unexpected complications have arisen in some cases (Alves et al., 2012). An oral drug that represents an alternative to azoles is 5-flurocytosine (5-FC), although there are concerns regarding resistance and toxicity associated with high doses used to negate resistance. Surprisingly, 5-FC/azole combinations have been reported to have an antagonistic effect in *C. glabrata* (Te Dorsthorst et al., 2002; Alves et al., 2012). This antagonistic effect seems to be mediated through the Pdr pathway as it was found that this phenomenon was abrogated in *Cgpdr1*Δ and *Cgcdr1*Δ strains (Steier et al., 2013). 5-FC exposure resulted in 6-fold induction of CgCdr1 that was CgPdr1-dependent even though 5-FC is not a CgCdr1 substrate. Interestingly, 5-FC exposure induced high frequency formation of petite (ρ^0^) mutants that upregulate CgPdr1-dependent CgCdr1 activation. Though the molecular mechanism contributing to this antagonistic effect is not clearly understood, it seems that the mitochondrial retrograde signal that activate CgPdr1 is the likely basis for this phenomenon in *C. glabrata*.

### Other pathways of multidrug resistance

As mentioned previously for *C. albicans*, *C. glabrata* also contains a number of MFS-encoding genes. While function of these membrane transporter proteins remains to be characterized, a *C. glabrata* homolog of ScFlr1 has been investigated for its potential role as a multidrug resistance determinant (Chen et al., 2007). The *C. glabrata* homolog was designated *CgFLR1* and as in both *C. albicans* and *S. cerevisiae*, disruption mutations lacking this gene did have drug phenotypes but were unaffected in terms of fluconazole susceptibility. *CgFLR1* is regulated by the *C. glabrata* homolog of ScYap1 (CgAP1) but this regulation does not appear to be involved in normal azole tolerance. More recent experiments have demonstrated that additional MFS proteins contributed to multidrug resistance including CgTPO3 and CgQDR2 (Costa et al., 2013, 2014).

## Multidrug resistance in *Aspergillus fumigatus*

Significant increases in immunocompromised population over the past few decades have resulted in a concomitant increase in invasive aspergillosis, with *Aspergillus fumigatus* being the most common causative agent (Denning et al., 2013a,b). Invasive aspergillosis is associated with a high rate of morbidity and mortality (as high as 50%) in these patients (Nivoix et al., 2008). However, there are limited effective antifungal drugs available to treat invasive apergillosis (see Pound et al., 2011 for a review). Though amphotericin B has been used with some success, it has been associated with high nephrotoxicity. Echinocandins such as caspofungin have been effective only as a topical agent and has been at best fungistatic. The only oral and the most widespread treatment against invasive aspergillosis has been triazole drugs, particularly itraconazole and voriconazole. However, continuous use of triazoles has led to the development of resistance against different azole drugs used for therapy, with clinical instances of such drug resistance increasing significantly in the last decade.

A prevalent mechanism that is associated with multiazole resistance against *A. fumigatus* has been alterations in the levels of the target enzyme, 14α-sterol demethylase, encoded by *cyp51A* gene (that also happens to have a paralog in *A. fumigatus*, namely *cyp51B*). The L98H mutation in the *cyp51A* gene linked with a 34bp tandem repeat sequence in its promoter has been associated with a large number of multi-azole resistant *A. fumigatus* clinical isolates (Snelders et al., 2008). This mutation was first reported in the Netherlands (where use of azoles as fungicides in agriculture is common), and was later reported from different parts of the world, indicating the spread of this mutation (Lockhart et al., 2011; Camps et al., 2012a; Chowdhary et al., 2012). Other mutational hotspots in *cyp51A* associated with drug resistant isolates have also been identified, though they have been less common and widespread (Recently reviewed in Lelievre et al., 2013).

Since 2008, a large proportion of multi-azole resistant *A. fumigatus* clinical isolates have been shown to be non-*cyp51A* dependent, implicating the role of other drug resistance mechanisms. A comprehensive study involving 64 azole resistant *A. fumigatus* strains done in the United Kingdom revealed that 43% of the cases had no *cyp51A* mutations associated with them (Bueid et al., 2010). Subsequent studies have identified that non-*cyp51A* based drug resistance is on the rise, with more than 50% of the cases involving alternate mechanisms (Camps et al., 2012b; Escribano et al., 2013). Analysis of these non-*cyp51A* based drug resistance isolates have revealed overexpression of ABC transporters in many of these cases, particularly that of cdr1B (abcB) (Fraczek et al., 2013).

Overexpression of efflux pumps is a common and well documented mechanism involved in the drug resistance of pathogenic yeasts such as *C. albicans* and *C. glabrata* as discussed above. They involve two types: the ABC class and the MFS class. The genes encoding these transporters [around 50 ABC (Kovalchuk and Driessen, 2010) and nearly 300 MFS class] are highly redundant in *A. fumigatus*, and some of them have been implicated in multi-azole resistance in *A. fumigatus* in recent years (Slaven et al., 2002; Nascimento et al., 2003; Ferreira et al., 2005). Among the transporters so far characterized in drug resistant clinical isolates of *A. fumigatus*, *cdr1B* (*abcB*) levels has been found to be most consistently and prominently overexpressed, with *cdr1B* mRNA transcript levels induced between 5–30 fold in these strains. Deletion of *cdr1B* also resulted in a 4-fold increase in susceptibility to itraconazole in both drug susceptible and resistant strains (Fraczek et al., 2013). In an independent study, the *abcB* gene was disrupted in three different *A. fumigatus* genetic backgrounds, resulting in susceptibility to different azoles tested: voriconazole, itraconazole as well as ketoconazole (Paul et al., 2013). Deletion of *abcA*, another ABCG class transporter like *abcB*, also resulted in drug sensitivity, at least in two different *A. fumigatus* genetic backgrounds, although to a lesser extent. Interestingly, overexpression of abcA led to an enhanced tolerance to azole drugs. This study also demonstrated that both *abcA* and *abcB* promoters are induced in the presence of drugs, just like in the case of *CgCDR1*. The action of efflux pumps (AfuMDR4) has also been implicated in fungal resistance within the biofilm, especially in CF patients (Rajendran et al., 2011).

As of yet, we know very little of the identity of transcriptional regulatory proteins involved in control of multidrug resistance in *A. fumigatus*. Analysis of a truncated Afyap1 (*A. fumigatus* homolog of ScYap1) determined that this mutant protein led to antifungal drug resistance (as found for truncated ScYap1-based drug resistance in yeasts), along with conferring tolerance to oxidative stress. However, multiple copies of full-length Afyap1 exhibited voriconazole susceptibility comparable with that of a wild-type *A. fumigatus* strain (Qiao et al., 2010). Together, these data suggest that Afyap1, like the bZip cognate proteins in yeast, exerts effects on antifungal drug resistance only upon mutational activation.

### Prospectus

The limited repertoire of antifungal drugs makes developing new modalities an important and pressing need. However, a goal of crucial importance to the enterprise of antifungal therapies in particular and drug-based therapies in general is to understand mechanisms of drug resistance. Multidrug resistance is an especially serious issue as a small number (often one) of genetic changes can lead to loss of susceptibility to multiple drugs simultaneously. In terms of antifungal multidrug resistance, our most developed picture applies to the yeast *Saccharomyces cerevisiae*, due in large part to the facile genetics of this organism. In recent years, important progress has been made in the two major pathogenic *Candida* species, *C. albicans* and *C. glabrata*. While there are significant similarities between the *Candida* species and *S. cerevisiae*, important differences have been described. The increasing development and application of genetic technologies in the *Candida* species will expedite direct study of the resistance mechanisms in these organisms. This is crucial to ensure continued utility of existing and future antifungal drugs.

While we have a fairly extensive catalogue of genes that are likely participants in the process of multidrug resistance, our understanding of how these genes are regulated is still very incomplete. In some cases, the identities of the transcription factors that control the multidrug resistance genes are well-established; yet a detailed picture of how the activity of these factors is regulated is not available. This will be an important future goal as interference with regulation of multidrug resistance gene expression has promise as a potential avenue to prevent development of this phenotype.

Improvements in medical care in regards to longer term survival in the face of immunosuppression has led to a greater chronic reliance on antimicrobial chemotherapy. *Candida* bloodstream infections are now the 4th most common form of nosocomial infection seen (Lewis, 2009). The occurrence of aspergillosis is also on the rise and outcomes associated with azole tolerant forms of this filamental fungal pathogen are dramatically worsened compared to azole susceptible fungi (Van Der Linden et al., 2011). Continued progress in the molecular understanding of antifungal drug resistance is an crucial step toward ensuring the sustainability of antifungal drug therapy and confidently expecting to treat these microbial pathogens in the future.

### Conflict of interest statement

The authors declare that the research was conducted in the absence of any commercial or financial relationships that could be construed as a potential conflict of interest.
